# Coping strategies of Dutch servicemembers after deployment

**DOI:** 10.1186/s40779-019-0199-4

**Published:** 2019-04-01

**Authors:** Loes G. M. de Kruijff, Olivia R. M. Moussault, Marie-Christine J. Plat, Rigo Hoencamp, Peter van der Wurff

**Affiliations:** 1 Department of Research and Development, Military Rehabilitation Center Aardenburg, Doorn, 3941 PW The Netherlands; 2 De Hoogstraat Rehabilitation, Utrecht, 3583 TM The Netherlands; 3 Military Mental Healthcare, Ministry of Defense, Zwolle, 8022 AE, The Netherlands; 4 Force Health Protection, Expert Center Force Health Protection, Ministry of Defense, Doorn, 3941 PW The Netherlands; 5 Department of Surgery, Leiden University Medical Center, 2333 ZA Leiden, The Netherlands; 6 Department of Surgery, Alrijne Medical Center, Leiderdorp, 2334 CK The Netherlands; 7 Department of Surgery, Central Military Hospital Ministry of Defense, Utrecht, 3584 EZ, The Netherlands; 8 Institute of Human Movement Studies, HU University of Applied Sciences Utrecht, Utrecht, 3584 CS The Netherlands; 9 Department of Research and Development, Military Rehabilitation Center Aardenburg, P.O. box 185, Doorn, 3940 AD The Netherlands

**Keywords:** Military personnel, Mental health, Coping behavior, Afghanistan, Rehabilitation

## Abstract

**Background:**

This study examines the relationship between coping strategies and symptoms of anxiety or depression among Dutch servicemembers deployed to Afghanistan.

**Methods:**

Coping strategies were assessed in 33 battlefield casualties (BCs) and the control group (CTRLs) of 33 uninjured servicemembers from the same combat units using the Cognitive Emotion Regulation Questionnaire. A factor analysis was performed, and two clusters of coping strategies were derived, namely, adaptive and maladaptive coping. Symptoms of anxiety and depression were evaluated using the depression and anxiety subscales of the Symptom Checklist-90-Revised. Correlations between coping and symptoms of anxiety and between coping and symptoms of depression were calculated, and a logistic regression was performed.

**Results:**

A moderate correlation was observed between maladaptive coping and symptoms of anxiety in the BC group (*r* = 0.42) and among the CTRLs (*r* = 0.56). A moderate correlation was observed between maladaptive coping and symptoms of depression in both groups (*r* = 0.55). The statistical analysis for the total sample (BCs and CTRLs) demonstrated no association between coping and symptoms of anxiety or depression.

**Conclusions:**

A correlation but no association was observed between maladaptive coping and mental health disorders in deployed Dutch servicemembers. Further research should focus on constructing cluster profiles of coping strategies and associating them with mental health outcomes and reintegration into society.

## Background

Combat exposure increases the risk of developing mental health disorders [1, 2]. The number of U.S. servicemembers who met the criteria of depression or anxiety disorder increased significantly after Operation Iraqi Freedom (OIF) and Operation Enduring Freedom (OEF) [3, 4]. The follow-up of Dutch servicemembers after Operation Task Force Uruzgan (TFU; 2006–2010) showed an increased risk of mental health disorders with a higher risk for those who operated predominantly off-base [5, 6].

Servicemembers who sustain combat-related injuries must cope with physical impairments and other stressors related to their injuries. Such individuals have a greater risk of developing mental health disorders than do their uninjured peers [7–9]. Battlefield casualties (BCs) from Operation TFU showed higher levels of depression and anxiety than those of uninjured servicemembers from the same combat units [10].

In the Dutch army, after repatriation to the Central Military Hospital, the majority of injured personnel are referred to the Military Rehabilitation Center Aardenburg (MRC). Rehabilitation programs focus mainly on enhancing participation in daily life. Improving participation, e.g., work and community reintegration, is more difficult for injured veterans with mental health problems [11, 12]. Veterans who perceive social support and use constructive coping strategies have better mental health outcomes than do veterans who use unconstructive coping strategies [13]. In the rehabilitation programs of the MRC, coping is not assessed.

Physicians practicing in physical medicine and rehabilitation agree that coping is an important factor of the outcome of a rehabilitation program. Survivors of a traumatic event experience a greater threat to one’s life when injured, especially in cases when they have less control over a situation. This diminished control results in higher levels of perceived stress. After sustaining combat-related injuries, servicemembers also have to confront additional stressors. Servicemembers suffer from physical and psychological consequences of their injuries, such as immediate repatriation, pain and lack of control over body functions. Regulation of emotions (coping) caused by these stressors plays an important role in posttraumatic adaptation [14].

The coping strategies individuals use when confronted with stress can affect both short-term and long-term physical and mental functioning. The problem, though, is that many coping strategies, as well as several classifications to categorize these coping strategies, have been described. In general, adaptive coping responses remove or lessen both fear and the danger of a threat and reduce stress levels. Maladaptive responses reduce the level of fear without reducing the danger, which increases stress levels and is associated with symptoms of depression or anxiety [15]. Adaptive coping, as opposed to maladaptive coping, improves outcomes, e.g., in physical health and social functioning [16–18].

Since coping is based on multiple factors, it is not plausible to assume that individuals use only a single coping strategy [19]. Managing trauma and its consequences, survivors may use more than one coping strategy. The focus in research is increasingly on coping profiles created by clustering coping strategies with regard to how individuals adapt [15, 20].

The aim of this study is to assess the relationship between clusters of coping strategies and symptoms of depression or anxiety in Dutch BCs from Operation TFU.

## Methods

### Study population

All Dutch servicemembers who suffered from combat-related injuries during Operation TFU (2006–2010) and underwent rehabilitation at the MRC Aardenburg, Doorn, the Netherlands, were included; none was excluded. Combat-related injury is specified as an injury incurred as a direct result of hostile action in combat or sustained while going to or returning from a combat mission [10]. The BCs were registered in the general digital admission database of the Dutch Ministry of Defense (MOD). The control group (CTRLs) consisted of uninjured servicemembers from the same combat units. The only exclusion criteria for this cohort was having incurred any injury, either combat-related or non-combat-related. The CTRLs were randomly selected by an independent epidemiologist from the section of Social and Behavioral Research of the MOD. They were matched by gender, age and rank during deployment.

All servicemembers were invited by post and email to complete an online questionnaire between December 2013 and July 2014. If necessary, they received two digital reminders and two reminders by telephone.

### Measurements

#### Participant characteristics

The following data were recorded: gender, age, marital status, number of deployments, educational level, and rank during deployment. Rank during deployment was divided into five rank groups: junior enlisted (E1-E4), senior enlisted (E5-E9), warrant officers (WO1-WO2), junior officers (O1-O3), and senior officers (O4-O10). The duration, in days, of the follow-up period after injury was recorded.

A previous study of the same cohort of Dutch servicemembers with combat-related injuries showed that almost all injuries were caused by explosions (47/48). The average number of injuries per servicemember was 5.2, and the majority of these injuries were located at the extremities [21].

#### Cognitive coping

The Cognitive Emotion Regulation Questionnaire (CERQ) is a multidimensional questionnaire constructed to identify the cognitive coping strategy someone practices after experiencing a negative event. The questionnaire measures nine different coping strategies: positive reappraisal, self-blame, positive refocusing, catastrophizing, putting in perspective, refocus on planning, rumination, acceptance, and blaming others [22]. Positive reappraisal, positive refocusing, perspective-taking, planning and acceptance are examples of adaptive coping. Rumination, catastrophizing and blaming others are examples of maladaptive coping [15, 23]. The coping strategy of self-blame was left out because it could indicate an internal locus of control (behavioral self-blame) with an adaptive effect, or an external locus of control (characterological self-blame) with a maladaptive effect [24]. For the purpose of this research, the CERQ-short questionnaire, a derivative of CERQ consisting of 18 items [25], was used.

#### Depressive and anxiety symptomatology

To assess mental health problems, the Dutch language version of the Symptom Checklist-90-Revised (SCL-90-R) was used. SCL-90-R is a widely used self-reporting instrument for assessing psychosocial distress. It includes 90 questions rated on a 5-point scale, with higher scores meaning greater psychological distress. SCL-90-R is divided into nine symptom subscales: anxiety (range 10–50), depression (range 16–80), somatization (range 12–60), hostility (range 6–30), insufficiency (range 9–45), agoraphobia (range 7–35), sensitivity (range 18–90), sleeping disorder (range 3–15), and additional items (range 9–45) [26, 27]. Depressive and anxiety symptomatology was measured using the depression and anxiety subscales of SCL-90-R.

### Data analysis

For data analysis, SPSS version 21.0 (SPSS Inc., Chicago, IL, USA) was used.

Factor analysis was used to define whether eight coping strategies (positive reappraisal, positive refocusing, putting into perspective, acceptance, refocus on planning, rumination, catastrophizing, and blaming others) could be divided into two groups. A principal component analysis was performed, followed by an orthogonal (varimax) rotation. The Kaiser-Meyer-Olkin test and Bartlett’s test of sphericity were used to assess if the data were suited for factor analysis. Due to the small sample size, no cut-off point was used for factor loading. The coping strategies were classified in one of the two groups based upon the highest factor loading. For each group, it was determined if coping strategies clustered in that group fit either the adaptive or maladaptive coping profile. The Kolmogorov-Smirnov test was performed to determine the normality of the distribution of scores.

Correlations between symptoms of anxiety or depression and the two groups of coping strategies were analyzed. The following limits were applied to interpret the strength of the association: *r* = 0–0.19 was regarded as very weak, 0.20–0.39 as weak, 0.40–0.59 as moderate, 0.60–0.79 as strong, and 0.80–1 as very strong correlation [28].

If the distribution of data was normal, a regression analysis was conducted; otherwise, a logistic regression analysis was performed to establish the association between coping as an independent variable, and symptoms of anxiety and depression as dependent variables. A linear relation between the variables is a prerequisite for logistic regression analyses. If no linearity was observed, coping and maladaptive coping were divided into quartiles. Rank and the number of deployments were added as confounders. The variable “sustaining injury” was used twice as an interaction term by multiplying it by adaptive and maladaptive coping. The interaction terms were added to assess whether there was effect modification [29].

### Ethical approval

The MOD, the Institutional Review Board and the Medical Ethics Committee of Leiden University, the Netherlands have approved this study (p11.184).

## Results

Fifty-eight servicemembers went through a rehabilitation program at the MRC, and 33 (57%) participated in the study. The mean follow-up period after incidence of BCs was 1925 days (interquartile range: 1349-2825). The demographics are summarized in Table 1.

**Table 1:** Demographics of battle casualties (BCs) and the control group (CTRLs)

Characteristics	BCs (*n* = 33)	CTRLs (*n* = 33)
Gender [*n*(%)]		
Male	31 (94)	31 (94)
Female	2 (6)	2 (6)
Age [year, mean ± SD)]	24.9 ± 6.8	26.3 ± 6.1
Marital status [*n*(%)]		
Single	10 (30)	6 (18)
In a Relationship	15 (46)	12 (36)
Married	7 (21)	15 (46)
Unknown	1 (3)	–
Educational level [*n*(%)]		
Low	1 (3)	1 (3)
Middle	25 (76)	28 (85)
High	6 (18)	4 (12)
Unknown	1 (3)	–
Rank during deployment [*n*(%)]		
Junior enlisted (E1-E4)	21 (64)	20 (61)
Senior enlisted (E5-E9)	8 (24)	9 (27)
Junior officers (O1-O3)	4 (12)	3 (9)
Senior officers (O4-O10)	–	1 (3)
Number of deployments [median (IQR)]	2 (2)^*^	2 (2)

*SD*. Standard deviation; *IQR*. Interquartile range; −. No data; ^*^. *N* = 31, the number of deployments for two BCs were missing

To assess if coping strategies could be divided into two groups, a principal component analysis with an orthogonal rotation was performed, dividing the sample into two groups based on the highest factor loading (Table 2).

**Table 2:** Principal component analysis with orthogonal rotation

Strategy	Adaptive	Maladaptive
Positive reappraisal	0.80	0.13
Positive refocusing	0.66	0.08
Putting into perspective	0.78	−0.06
Acceptance	0.68	0.37
Refocus on planning	0.76	0.31
Rumination	0.30	0.82
Catastrophizing	−0.16	0.83
Blaming others	0.24	0.48

The Kaiser-Meyer-Olkin measure of sample adequacy was 0.77, and Bartlett’s test of sphericity was significant (*P* = 0.00). The items clustering together on the same factor confirmed that one factor represented adaptive coping, and the other represented maladaptive coping. The coping strategies of positive reappraisal, positive refocusing, putting into perspective, acceptance, and refocus on planning corresponded to adaptive coping. The coping strategies of rumination, catastrophizing and blaming others corresponded to maladaptive coping. Cronbach’s alpha for adaptive coping was 0.82. Cronbach’s alpha for maladaptive coping was 0.58. Removing an item did not improve the overall reliability of the scale.

The Kolmogorov-Smirnov test showed that the distribution of data was not normal. The correlations measured using Spearman’s rank correlation coefficient between maladaptive and adaptive coping, and anxiety and depression are shown in Table 3.

**Table 3:** Correlations between variables measured using Spearman’s rank correlation coefficient

Variable	Maladaptive	Adaptive	Anxiety	Depression	Median	IR
Maladaptive		0.27	0.42^*^	0.55^**^	1.67	1.17
Adaptive	0.55^**^		0.02	0.09	2.90	1.05
Anxiety	0.56^**^	0.24		0.71^**^	1.20	0.6
Depression	0.55^**^	0.24	0.70^**^		1.13	0.63
Median	1.33	2.20	1.00	1.06		N/A
IR	0.67	1.85	0.15	0.22	N/A	

Intercorrelations for battle casualties (*n* = 33) are presented above the diagonal, and intercorrelations for the control group (*n* = 33) are presented below the diagonal. Median and interquartile range (IR) of battle casualties are presented in horizontal rows, and median and IR of the control group are presented in vertical rows. ^*^P < 0.05; ^**^P < 0.01

Since the data were not normally distributed, the scores of anxiety and depression were dichotomized such that a logistic regression analysis could be performed. Median scores were chosen for the cut-off point: for anxiety, 1.09 was chosen, and for depression, 1.12 was chosen. No linearity was observed between coping and symptoms of anxiety or depression; therefore, maladaptive coping and adaptive coping were divided into quartiles. The logistic regression analysis is shown in Table 4.

**Table 4:** Logistic regression analysis for anxiety and depression

Variable	Anxiety			Depression		
	*β*	Exp (*β*)	95% CI	*β*	Exp (*β*)	95% CI
*Unadjusted model*						
Adaptive						
Adaptive (1)	−0.73	0.48	0.07–3.23	0.49	1.63	0.26–10.0
Adaptive (2)	−0.67	0.51	0.08–3.36	0.48	1.63	0.26–10.21
Adaptive (3)	0.30	1.34	0.17–11.33	1.04	2.82	0.37–21.81
Maladaptive						
Maladaptive (1)	1.88	6.58	0.93–46.76	0.53	1.70	0.27–10.67
Maladaptive (2)	1.80	6.02	0.70–51.93	1.17	3.21	0.44–23.65
Maladaptive (3)	2.82	16.69	1.93–144.44	2.95	19.14	2.14–171.0
*Adjusted model* ^*^						
Adaptive						
Adaptive (1)	−0.69	0.50	0.07–3.40	0.50	1.65	0.27–20.12
Adaptive (2)	−0.52	0.60	0.08–4.25	0.74	2.09	0.30–14.55
Adaptive (3)	0.59	1.81	0.19–17.08	1.44	4.22	0.46–38.64
Maladaptive						
Maladaptive (1)	1.97	7.20	0.97–53.44	0.60	1.83	0.29–11.56
Maladaptive (2)	1.70	5.46	0.63–47.32	1.04	2.82	0.36–21.87
Maladaptive (3)	2.97	19.41	2.11–178.69	3.16	23.54	2.45–226.23

Adaptive. 1.0–2.0; Adaptive (1). 2.0–2,7; Adaptive (2). 2.7–3.4; Adaptive (3). 3.4–4.5

Maladaptive. 1.0–1.17; Maladaptive (1). 1.17–1.5; Maladaptive (2). 1.5–2.0; Maladaptive (3). 2.0–3.5; *.Model adjusted for the number of deployments and rank

The unadjusted model shows no association between adaptive coping and symptoms of anxiety or depression, and maladaptive coping and symptoms of anxiety or depression. Adding two confounders − rank and the number of deployments − affected the highest scores for adaptive coping in relation to anxiety. The confounders also affected the highest score for adaptive coping, and the middle scores for maladaptive coping in relation to depression. However, in the adjusted model, there is no association between coping and symptoms of anxiety, or coping and symptoms of depression (all *P* values were > 0.05) for the total sample (BCs and CTRLs).

## Discussion

A moderate correlation was observed between maladaptive coping and symptoms of anxiety and between maladaptive coping and symptoms of depression in BCs and in CTRLs. No association was observed between coping and symptoms of anxiety or depression.

Doron et al. adopted 3 clusters of coping strategies in the general population: adaptive, avoidant and low [23]. Smith et al. derived 4 ways of coping: individuals practicing active coping strategies, individuals practicing passive coping strategies, individuals practicing low coping strategies and individuals practicing self-blame [30]. The researchers suggested that individuals practicing active coping strategies showed adaptive coping skills, and individuals practicing passive coping strategies showed maladaptive coping skills. Compared to the studies of Doron et al. and Smith et al., individuals practicing low coping strategies showed low levels of coping strategies in general. Individuals practicing active coping strategies showed higher levels of positive reappraisal, positive refocusing, and putting into perspective, while individuals practicing avoidant coping strategies showed higher levels of self-blame, rumination, catastrophizing, and blaming others [23]. Individuals practicing adaptive coping strategies displayed lower levels of depression and anxiety than did individuals practicing avoidant or maladaptive coping strategies [23, 30].

There can be several reasons for the lack of an association between coping and symptoms of anxiety or depression, including a low sample size resulting in a low variability or low scores of depression and anxiety with a small spread of data. More importantly, the data had to be processed to perform a regression analysis. In an already low sample size, coping strategies had to be divided into quartiles, and scores of anxiety and depression had to be dichotomized. Dichotomization can result in a loss of effect size and statistical significance [31]. Studies with a larger sample size are required to assess if an association can be demonstrated.

Another reason for the lack of an association between coping and symptoms of anxiety or depression can be due to Cronbach’s alpha of 0.58 for maladaptive coping. This is a relatively low score according to current views; however, it is acceptable for lack of better options. The low Cronbach’s alpha can be due to several reasons: a low number of questions, or poor interrelatedness between items (due to excessive heterogeneity in the constructs) [32]. Only two questions represent one coping strategy, so the small number of questions could be one of the reasons for a low Cronbach’s alpha. The alternatives would be to use the full-scale CERQ consisting of 36 items instead of 18 items, or to construct more coping profiles (e.g., adaptive, maladaptive, and individuals practicing low coping strategies).

The low scores of anxiety and depression in BCs are remarkable. Many symptoms of depression and anxiety overlap with the symptoms of posttraumatic stress disorder (PTSD). Eekhout et al. reported that 9% of 1007 Dutch servicemembers had delayed onset of symptoms of PTSD 5 years after OEF with lower ranks (junior enlisted) being at greater risk. The level of deployment stressors was a moderator; a higher level of deployment stressors was related to a greater increase in symptoms of PTSD [5]. Explanations could be that different questionnaires were used (the self-rating inventory for posttraumatic stress disorder vs. the SCL-90-R depression and anxiety scales), or the injured servicemembers in our study might have received treatment for mental health problems during the interim years.

Before OIF and OEF, fewer diagnostic tests were done to explore mental health problems, but other longer follow-up studies of previous wars showed that rank- and combat-related injuries were associated with mental health problems [33]. Low scores of anxiety and depression in our study could be due to underreporting of mental health symptoms. Several factors can impede reporting mental health problems: the stigma associated with admitting mental health problems vs. a medical problem, lack of perceived need for treatment, lack of trust in mental health professionals, treatment beliefs, and the perceived inconvenience of undergoing additional evaluation [34, 35]. Our study was confidential and anonymized but not completely anonymous. Our questionnaires asked if the subjects preferred personal contact in case of mental health problems. None of the participants exercised this option, but it could have influenced their answers, since they could contact caregivers.

Since OIF and OEF, more attention has been paid to mental health problems. The importance of enhancing psychological resilience to withstand mental health problems has been emphasized. The definition adopted by the U.S. military health care providers is “resilience is the capacity to adapt successfully in the presence of risk and adversity.” The factors that promote resilience are divided into individual-level factors including positive coping, family-level factors, unit-level factors, and community-level factors [36]. Not all factors had strong evidence of contributing to resilience; however, this phenomenon implies that further research should concentrate on not only coping but also other factors.

To assess the result of the effectiveness of a program to develop coping skills, outcome measures can be mental health-related (mood or anxiety disorders) but could also be stated in terms of functioning. This possibility suggests evaluating coping in terms of the International Classification of Functioning, Disability and Health (ICF) model that is used as a framework in rehabilitation medicine practice, research and education. Rehabilitation programs aim to enhance and restore functional ability and quality of life to those with physical impairments or disabilities. The ICF framework describes functioning as being a complex interaction of a person’s health condition, environmental factors and personal factors. Although the component ‘personal factors’ has not yet been classified, it includes psychological resources that influence how disability is experienced by the individual. Coping can be considered as a personal factor and evaluated in terms of measuring the level of participation of servicemembers with different coping skills. Consequences of combat-related injury, such as trauma-related pain and lack of control over body functions, can trigger negative thinking and impede rehabilitation. Maladaptive coping can be addressed with education and/or forms of cognitive-behavioral therapy, e.g., cognitive restructuring, and mindfulness [37, 38].

Since OIF and OEF, many studies have been published on mental health in veterans. This study adds the use of cluster analysis to research of coping in this group. For future rehabilitation programs, it is recommended to assess coping strategies and the relationship with symptoms of depression and/or anxiety, as well as the level of participation.

## Study limitations

The low sample size was a major limitation; however, the response rate of nearly 60% was acceptable. From the beginning, it was known that the maximum number of BCs that could participate was 58, which affected the choice of our statistical methods. We categorized coping strategies in 2 clusters instead of a larger number, and limited the number of confounders in the logistic regression. This approach might have affected the results, but it is impossible to be certain.

Another limitation was the retrospective design of the study, including the timing of the questionnaires (5 years post-incident).

## Conclusions

A moderate correlation was observed between maladaptive coping and mental health disorders in a small sample size of deployed Dutch servicemembers. To better understand mental health problems, more attention should be paid to clusters of coping strategies and the relationships between coping and mental health and between coping and functional outcome.

## Abbreviations

BCs: Battlefield casualties
CERQ: Cognitive Emotion Regulation Questionnaire
CTRLs: Control group
ICF: International Classification of Functioning, Disability, and Health
MOD: Ministry of Defense
MRC: Military Rehabilitation Center Aardenburg
OEF: Operation Enduring Freedom
OIF: Operation Iraqi Freedom
PTSD: Posttraumatic stress disorder
SCL-90-R: Symptom Checklist-90-Revised
TFU: Task Force Uruzgan
